# The role of core strength in front crawl performance: a qualitative analysis of expert coaches’ perspectives

**DOI:** 10.3389/fspor.2026.1748612

**Published:** 2026-05-20

**Authors:** Jinjin Dai, Junjun Xu, Zicheng Dai

**Affiliations:** 1 Department of Sports, Zhejiang Wanli University, Ningbo, China; 2 School of Information Technology and Artificial Intelligence, Zhejiang University of Finance and Economics, Hangzhou, China; 3 Swimming Department, Zhejiang College of Sports, Hangzhou, China

**Keywords:** coach decision-making, elite swimming, qualitative interviews, threshold effect, training transfer, trunk function

## Abstract

While core strength is theorized to be crucial for swimming performance, empirical evidence remains conflicting, and the perspectives of high-performance coaches—key stakeholders in training prescription-are largely unexplored. This qualitative study examined the perceptions of eight expert Chinese swimming coaches (seven males, one female) regarding the role of core strength in front crawl performance and explored how and why they prescribe core-specific training. Semi-structured interviews were conducted, transcribed verbatim, and analyzed using thematic analysis. Results revealed three central themes. First, all coaches unanimously affirmed core strength's importance through dual mechanisms: direct (force transmission, rotational power) and indirect (stabilization, drag reduction). Second, despite this consensus, assessment and training methods relied primarily on experiential knowledge rather than evidence-based protocols. Third, a “threshold effect” was identified, wherein coaches' experiential knowledge suggested that core training benefits may be most apparent in athletes with initial deficiencies. Based on these findings, we propose a conceptual model positing that core function operates through direct and indirect pathways, moderated by baseline capacity and transfer effectiveness. The model provides a testable framework to guide future research on how, when, and for whom core training is most effective, underscoring the need for swimming-specific diagnostic tools and integrated training protocols.

## 1 Introduction

The pursuit of elite athletic performance is fundamentally a multidisciplinary endeavor, reliant on the integrated efforts of sport scientists, athletes, and coaches to achieve both short-term and long-term objectives (1, 2). In the domain of competitive swimming, while the propulsive roles of the upper and lower limbs in front crawl have been extensively studied (3, 4), the contribution of core strength has received comparatively less empirical attention. This is despite its theorized significance in stabilizing the body, facilitating inter-segmental force transfer, and maintaining optimal hydrodynamic alignment.

Proper alignment of the head, shoulders, trunk, pelvis, and lower limbs forms the technical cornerstone of efficient swimming, as a streamlined body position minimizes hydrodynamic resistance (5, 6). The trunk musculature is considered crucial for maintaining this posture and providing active stabilization in the unstable aquatic environment (7). Insufficient core strength is thought to lead to energy leakage through compromised stabilization (8, 9), while a stable torso is deemed essential for optimising power output from the limbs (5). From a theoretical standpoint, training these muscles is believed to enhance stabilization, thereby increasing force production and improving the efficiency of force transmission from the core to the extremities (10). In sprint swimmers, such training may further induce beneficial neuromuscular adaptations that directly enhance stroke efficiency (10, 11). Hibbs et al. (10) proposed that core training enhances the efficiency of the kinetic chain by improving the timing and magnitude of muscle activation patterns, while Weston et al. (11) demonstrated that a six-week core training program improved sprint performance in national-level junior swimmers, suggesting transfer of neuromuscular adaptations to stroke mechanics.

However, the empirical evidence supporting a direct impact of trunk strength on swimming performance is conflicting. For instance, while core training has been shown to improve swimming efficiency in adolescents (12), studies on elite athletes often demonstrate minimal transfer to performance (13, 14). This discrepancy may stem from methodological limitations; many assessments rely on isometric or endurance-based tests that are ill-suited to capture the velocity-specific strength adaptations critical for sprint swimming. Furthermore, the role of the core in a stroke dominated by transverse-plane rotation, such as front crawl, is not fully understood. Some research suggests torso muscles are more pivotal for postural stability and control than for generating rotation itself (15), indicating that swimming performance may depend more on the technical integration of movement patterns than on isolated trunk strength. This body of conflicting evidence points to a fundamental gap: we lack a clear understanding of how, when, and for whom core strength contributes to swimming performance. This gap is not merely academic-it has direct implications for how practitioners prescribe core training and how sport scientists design meaningful interventions.

Existing investigations into core function have predominantly been approached through biomechanical and physiological lenses, employing largely quantitative methodologies (16). While quantitative studies have generated valuable but conflicting data on core muscle activation and its correlation with swim speed [e.g., (12–14)], their applicability is often constrained by controlled laboratory conditions that do not reflect the dynamic complexity of daily coaching practice. This limitation is particularly significant because coaches are the primary agents who translate-or fail to translate-scientific evidence into daily practice. Their decisions about what to train, how to assess it, and when to intervene are shaped by years of experiential learning and reflection, producing a rich blend of declarative and procedural (tacit) knowledge (17, 18). This reliance on practitioner knowledge, however, remains largely unexamined in the literature.

Qualitative inquiry offers a complementary lens that is uniquely suited to bridge this theory-practice gap. By capturing the nuanced, experience-based perspectives of practitioners, it can illuminate how core strength is conceptualized, diagnosed, and trained within the real-world constraints and affordances of the training environment (19, 20). Such insight is essential for understanding the “why” behind coaching decisions-a dimension that quantitative measures alone cannot capture. Moreover, understanding how expert coaches conceptualize the role of core strength can assist support staff in several key areas: interpreting and conveying scientific evidence, facilitating knowledge dissemination within the sport science community, and guiding future applied research agendas (21). Expert coaches, honed by years of practical experience and reflection, may possess insights that have not yet been validated-or even investigated-by empirical research. Surfacing this knowledge is therefore not merely supplementary but fundamental to constructing an ecologically valid theoretical framework that can directly inform and refine training prescription.

To date, the small body of qualitative research on strength and conditioning in swimming has focused primarily on general training practices rather than core-specific approaches. For instance, Morris et al. (4) explored expert coaches’ perspectives on the role of upper and lower limbs in front crawl, revealing nuanced understandings of limb coordination but not addressing core function specifically. More recently, Raineteau et al. (13) examined training and testing practices of strength and conditioning coaches working with French sprint swimmers, finding that core training was commonly prescribed but that assessment methods varied widely and lacked standardization. However, this study focused on the broader strength and conditioning landscape rather than providing an in-depth exploration of coaches’ conceptualizations of core function *per se*. To the best of our knowledge, no qualitative study has systematically investigated how expert swimming coaches conceptualize the role of core strength, assess core function in their athletes, and design core-specific training interventions. The present study is therefore exploratory and unique in its focused, qualitative examination of coaches’ core strength-related knowledge and practices.

The primary purpose of this study was therefore to examine the perceptions of expert high-performance swimming coaches regarding the influence of core strength on front crawl performance. Specifically, we aimed to explore: (1) how coaches conceptualize the role of core strength in front crawl performance; (2) how they assess core strength in practice; and (3) what methodologies they employ for core strength training. Given the exploratory, qualitative nature of this inquiry, we did not formulate *a priori* hypotheses in the hypothetico-deductive sense. However, consistent with the constructivist orientation guiding this study (22), we approached the research with certain sensitizing concepts-theoretical ideas that provide a starting point for inquiry without pre-determining findings. Specifically, we anticipated that coaches would: (1) hold nuanced, experience-based understandings of core function that extend beyond the mechanistic accounts prevalent in the literature; (2) employ assessment methods that prioritize ecological validity (in-water observation) over standardized but decontextualized tests; and (3) design training programs that reflect an implicit understanding of specificity and transfer, even if not formally articulated in scientific terms. These expectations were informed by the research team’s embeddedness in high-performance swimming contexts and by pilot discussions with coaches. The analysis remained open to findings that challenged or refined these initial expectations. In this study, ‘core strength’ refers to the capacity to generate and transmit force; ‘core stability’ refers to maintaining alignment under load or fatigue; ‘core function’ is used as an umbrella term encompassing both strength and stability, as well as coordination and transfer. These operational definitions are derived from coaches’ usage and consistent with Hibbs et al. (10).

## 2 Methods

### 2.1 Participants

This study employed purposeful sampling to recruit eight expert high-performance swimming coaches (seven males, one female) (22). While recognizing the limitations of using athletic outcomes as the sole criterion for coaching expertise, all participants met the “expert” threshold by virtue of having coached at least one Olympic medalist or having served as a National Head Coach in swimming. The coaches had an average of 21.5 ± 6.5 years of experience working with national-level swimmers, and all were currently or had previously been involved in coaching athletes competing at Olympic or world championship levels.

Beyond their coaching credentials, all coaches also possessed competitive swimming backgrounds themselves, with six specializing in front crawl, one in breaststroke, and one in butterfly. Their coaching experience encompassed all competitive distances-sprint (50–100 m), middle-distance (200–400 m), and distance (800–1,500 m)-with several coaches noting particular areas of emphasis (e.g., C1 and C5 specialized in sprint events; C3 had extensive experience with distance swimmers). This diversity of distance-specific expertise enriches the dataset by capturing potential variations in how core function is perceived across different event demands. The underrepresentation of female coaches (*n* = 1) reflects the gender composition of high-performance swimming coaching in the Chinese context, where male coaches currently predominate at the Olympic and national team levels; this limitation is acknowledged in the Discussion. Prior to the interviews, all participants provided signed informed consent, and the study protocol was approved by the Human Research Ethics Committee of Beijing Sport University.

The richness of this dataset is particularly noteworthy given the relatively small sample size-a decision guided by the concept of information power (23) rather than statistical power. Consistent with qualitative research principles, the study's narrow aim (core strength in front crawl), the highly specific sample (expert coaches with Olympic-level experience), the strong quality of interview dialogue, and the focused analytic strategy (thematic analysis) collectively ensured that rich, relevant data could be obtained from a relatively small number of participants.

Data saturation-defined as the point at which subsequent interviews yielded no new themes or insights-was assessed iteratively during data collection. Following each interview, the first author conducted preliminary coding and then discussed emerging patterns with the research team. This ongoing analysis revealed that the seventh interview produced no novel themes beyond those identified in the first six; the eighth interview was conducted to confirm saturation and similarly introduced no new concepts. Thus, data saturation was achieved with eight participants, with the final interview serving as confirmation.

### 2.2 Research design

**Figure 1:**
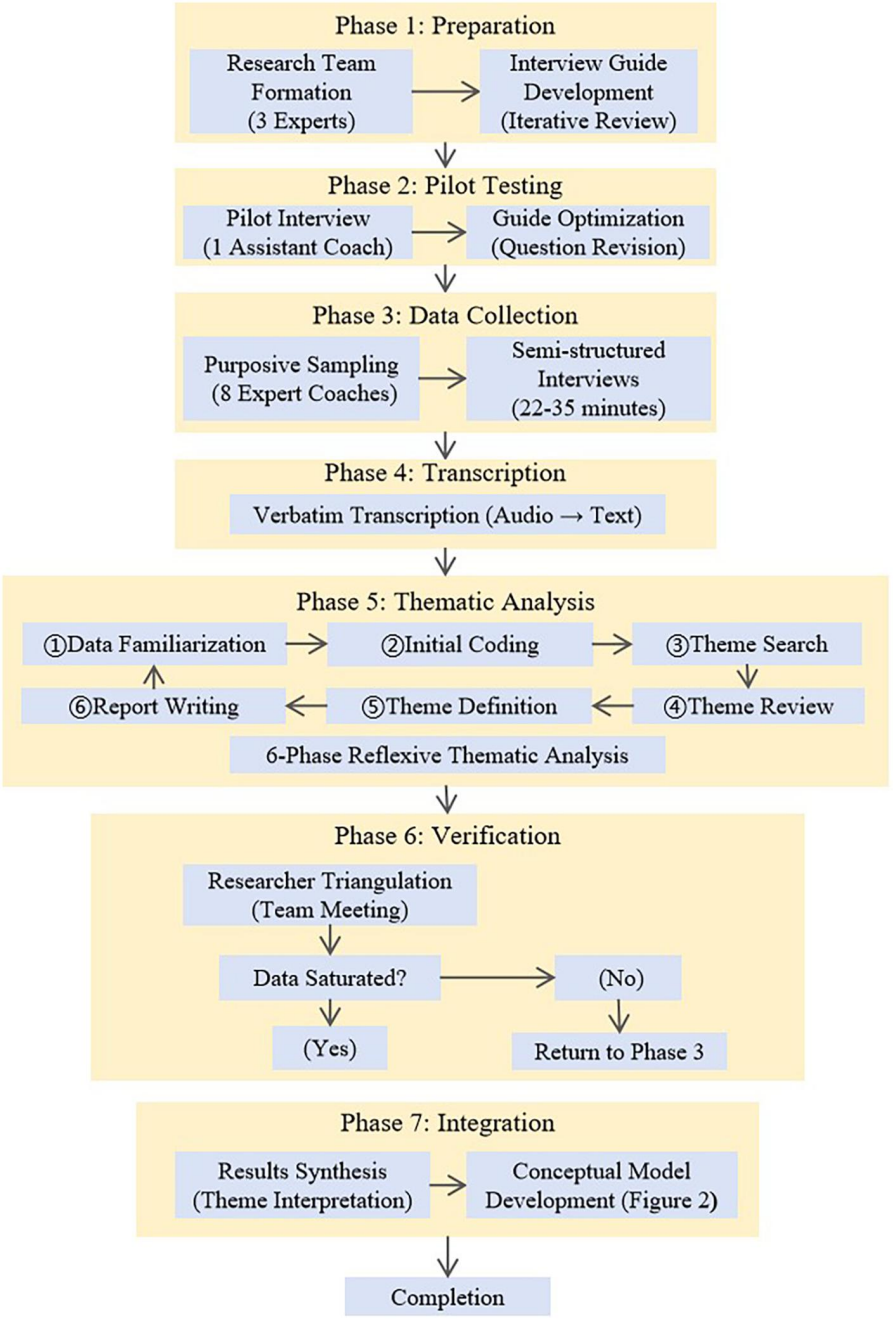
Study procedure flowchart illustrating the seven phases of the research process, from preparation through pilot testing, data collection, transcription, reflexive thematic analysis, and verification. The iterative feedback loop following saturation assessment is indicated, demonstrating how data collection continued until no new themes emerged.

A semi-structured interview protocol was developed by a panel of three specialists in sports pedagogy and swimming coaching. The panel reviewed and refined an initial pool of potential questions through an iterative process to ensure content validity and relevance to coaching practice. The final protocol consisted of eight open-ended questions (see Table 1) designed to explore coaches’ expert knowledge of core strength in swimming (24). It is important to clarify that, consistent with semi-structured interviewing methodology, these questions served as a flexible guide rather than a fixed script. While the interviews followed a consistent thematic progression, the sequence and precise wording of questions were adapted based on individual responses to maintain conversational flow and allow exploration of emergent topics. This flexibility enabled the interviewer to probe deeply into coaches’ reasoning, seek clarifications, and elicit concrete practical examples (22). The complete study procedure, from preparation through to analysis and model development, is illustrated in Figure 1 below. This flowchart provides a visual overview of the systematic process employed, including the iterative nature of data collection and analysis.

**Table 1:** List of interview questions.

No.	Question
1	What is the definition of “core” in core strength in swimming?
2	How does the core strength transmit the strength of upper and lower limbs in front crawl?
3	How does core strength play a role in technical movements in front crawl?
4	Are there significant differences in core strength among front crawl swimmers at different levels?
5	Is there a correlation between core strength and front crawl performance?
6	What are the core strength deficiencies in front crawl events at different distances?
7	In practice, how do you assess core strength?
8	In practice, what methods do you often use to strengthen core strength?

A pilot interview was conducted with an assistant coach from a high-performance program affiliated with one of the participants. Feedback from this pilot was used to refine the interview guide, particularly clarifying the phrasing of questions related to technical movement analysis. All formal interviews were carried out by the corresponding author, a fitness coach with extensive experience in physical conditioning for elite swimmers, including those who have medaled at the Tokyo Olympic Games and World Championships. This background was instrumental in several respects: it facilitated rapid establishment of rapport and credibility with the expert coach participants; it enabled the interviewer to understand and respond appropriately to technical terminology; and it enriched the contextual interpretation of the data during analysis (22). To mitigate potential bias arising from this insider perspective (see Researcher Positionality section), systematic safeguards were implemented.

Interview topics encompassed the definition of the core, its role in kinetic chain integration and technical execution, level-based differences, performance correlations, common weaknesses, assessment techniques, and training methodologies. Interviews ranged from 22 to 35 min (median = 25.5 min). The focused nature of the interview guide, combined with coaches’ direct expertise on the topic, enabled rich information to be obtained within these timeframes, which were constrained by coaches’ demanding schedules. Seven were conducted face-to-face in locations chosen by the participants, and one was held via telephone. All interviews were audio-recorded by the corresponding author, and verbatim transcripts were prepared and initially coded by the first author.

### 2.3 Data analysis

Adopting a constructivist orientation (22), the interview transcripts were analyzed using reflexive thematic analysis following the six-phase approach outlined by Braun and Clarke (25). This framework was selected for its theoretical flexibility and its emphasis on the active role of the researcher in knowledge production. The analysis began with data familiarization, during which the lead researcher immersed themselves in the data through repeated readings of transcripts while listening to audio recordings, noting initial impressions and potential patterns. This was followed by sentence-by-sentence open coding, which generated 78 initial codes capturing both semantic and latent meaning in the data. This initial coding phase was completed independently by the first author to ensure that the coding process was not unduly influenced by the second author's insider perspective. Codes were then grouped into provisional categories based on conceptual similarities, forming candidate themes. These candidate themes were subsequently reviewed against coded extracts and the entire dataset to ensure they accurately represented the data; axial coding techniques were employed at this stage to explore relationships between categories and refine the thematic structure (26). Throughout phases three to five, regular research team meetings were held to critically discuss interpretations, challenge assumptions, and reach consensus on the thematic structure-a process of investigator triangulation that enhanced the trustworthiness of the analysis by subjecting interpretations to multiple perspectives. Finally, themes were defined and named, with each theme's scope and content clearly articulated before being finalized and presented in the Results section with representative quotations from coaches (C1–C8), in line with qualitative reporting standards (24).

### 2.4 Trustworthiness and rigor

Several strategies were employed to enhance the trustworthiness of the findings. Credibility was established through prolonged engagement with the data, investigator triangulation via team meetings, and the inclusion of rich, verbatim quotations that ground interpretations in participants’ own words. Dependability was ensured by maintaining an audit trail that documented analytical decisions, coding frameworks, and theme development throughout the research process. Confirmability was supported through reflexive practices and independent initial coding by the first author, which helped ensure that findings emerged from the data rather than from researcher preconceptions. Transferability was addressed by providing thick description of the participant sample, context, and analytical procedures, enabling readers to assess the applicability of findings to other settings. While member checking was not formally conducted, the research team determined that the iterative verification achieved through team discussions and the attainment of data saturation provided sufficient rigor for this exploratory study; nevertheless, we acknowledge the absence of member checking as a potential limitation.

### 2.5 Researcher positionality and reflexivity

The corresponding author is a fitness coach with extensive experience in elite swimming conditioning-a background that was crucial for establishing rapport with participants and contextualizing the data during both interviews and interpretation. However, this insider perspective also carried potential for bias, specifically the risk of imposing preconceived understandings or failing to probe taken-for-granted assumptions. To mitigate this risk, several safeguards were implemented. First, the first author, who was not directly involved in elite swimming coaching, conducted the initial independent coding, thereby providing an external perspective on the data. Second, during team meetings, the second author explicitly reflected on how their experiential knowledge might shape interpretation, and these reflections were critically examined by the broader research team. Third, all interpretations were subjected to team discussion, with alternative readings actively solicited and considered before consensus was reached. Finally, an audit trail documented reflexive notes and analytical decisions throughout the research process, ensuring transparency. All interview data were anonymized and stored securely to maintain confidentiality.

## 3 Results

Thematic analysis of the interview transcripts revealed three central themes concerning the role of core strength in front crawl swimming: (a) the perceived function of the core in enhancing performance, (b) current practices in assessing core strength, and (c) methodologies for core strength training. Each theme is substantiated with direct quotations from the coaches (C1–C8), providing illustrative evidence of the emergent categories. Where appropriate, we provide interpretive commentary to elucidate the underlying reasoning and implications of coaches’ statements.

### 3.1 Theme 1: the functional role of the core in swimming performance

#### 3.1.1 Conceptualization of core strength

Coaches exhibited a nuanced understanding of core strength, with definitions varying in scope and emphasis. These variations reflect not merely semantic differences but distinct conceptual frameworks that shape how coaches approach training prescription.

C6 highlighted *the shoulder girdle and hip complexes as primary force generators, stressing the importance of bilateral symmetry*. This emphasis on symmetry suggests a concern with preventing compensatory patterns that could lead to injury or inefficiency. C4 defined the core anatomically as “*the region from below the shoulders to above the knees*,” with functional emphasis on hip-driven movement-a definition that prioritizes the role of the core in linking lower-body power generation to upper-body force application.

C2 offered a more integrated view, *describing the core as the critical link between the upper and lower limbs, essential for whole-body coordination*. This conceptualization positions the core as a conduit rather than a generator-a distinction with important implications for training design. C1 further differentiated core function by stroke type, noting that *while butterfly and breaststroke rely on active trunk flexion-extension, front crawl and backstroke depend on rotational stability and control*. This stroke-specific conceptualization demonstrates how expert coaches tailor their understanding of core function to the unique demands of different events.

Despite these conceptual differences, all coaches regarded the core as fundamental to technical efficiency and performance. The variation in conceptualization should therefore be understood not as disagreement but as complementary perspectives that together constitute a rich, multidimensional understanding of core function.

#### 3.1.2 Mechanisms of performance enhancement

Coaches identified multiple pathways through which core strength influences front crawl performance, which can be broadly categorized into direct and indirect effects. The identification of dual mechanisms is significant because it suggests that core training may need to address qualitatively different functions, potentially requiring distinct training modalities.

### 3.2 Direct contribution to propulsion

Some coaches asserted that core strength directly enhances sports performance by boosting driving force, citing athletes whose swimming speeds improved noticeably with enhanced core strength. The reasoning underlying this claim warrants examination: swimmers lack fixed support in water, making core strength essential for effective limb movement and propulsion. Some coaches employed a boat analogy, comparing a stable trunk to a hull that facilitates maximum propulsion-a mental model that frames core function as enabling rather than generating.

Swimmers lack fixed support in water, making core strength essential for effective limb movement and propulsion. Some coaches compare an athlete's body to a boat, where a stable trunk, akin to a hull, facilitates maximum propulsion.

Initially, these athletes, at first-level or higher, exhibited weaknesses in various strength types. During their training, they focused on exercises like jumping, dynamic running, sit-ups, multi-directional static holds, and basic yoga ball routines, which constituted their primary land training regimen. This training enabled them to better control their bodies and coordinate force exertion in water, leading to significant improvements in their sports performance and swimming speed, particularly in long-distance events. Comparative test data from before and after the winter training period demonstrated a marked increase. (C3)

Coaches concurred that the trunk acts as the pivotal force-transfer point in front crawl, with its rotational strength critically interlinked with performance. C7 emphasized this linear relationship, stating mastery of resultant force yields significant gains. This is exemplified by elite swimmers who effectively utilize axial rotation-a skill C6 tied directly to core control-to minimize drag and enhance propulsion, in contrast to less skilled swimmers who over-rely on upper-limb strength.

The coordination of opposite-side hands and feet in front crawl ensures balance and stability, with shoulder girdle muscle contraction being the primary source of propulsion. Only the shoulders and hips engage in horizontal axis rotation, effectively transmitting power to the extremities. (C6)

These quotations collectively reveal how coaches conceptualize the core's direct role: C3 articulates a logic chain from enhanced core control to improved performance, grounded in observable outcomes, while C6 specifies horizontal axis rotation as the key mechanism, positioning the core as a conduit-rather than a generator-of power.

### 3.3 Indirect role through stabilization and drag reduction

The majority of coaches emphasized the core's role in maintaining hydrodynamic alignment and reducing resistance.This indirect pathway was described with considerable specificity, suggesting that coaches have developed detailed mental models of how core insufficiency manifests in observable technical flaws.

C5 explained that fatigue-induced “waist collapse” leads to dropped hips and increased frontal drag, particularly in sprint events. The mechanism described here is fatigue → loss of core integrity → postural deterioration → increased drag → reduced speed. This causal chain has clear implications for training: if core failure under fatigue is the problem, then core endurance and fatigue resistance become critical training targets.

During repeated 50 m sprints, athletes experience fatigue, particularly in the waist, hips, and legs, leading to a noticeable drop in these areas. This waist collapse can lower the leg position, creating an “uphill swimming” sensation. The increased resistance from this posture is primarily due to trunk fatigue, which disrupts technical stability. In front crawl, this results in a wider lateral profile, while in butterfly, it increases the vertical profile, ultimately slowing the swimming speed. (C5)

C1 added that inadequate trunk stability compromises limb kinetics and technical consistency, especially during the final stages of races. This observation extends the logic further: core failure not only increases drag directly but also forces compensatory limb actions that further degrade efficiency.

In swimming, a collapsing waist increases pressure at higher speeds, generating vortices that intensify with speed. A collapsed waist requires greater limb effort for control and compensation, diminishing the swimmer’s ability to maintain speed. Given identical conditions, stronger core strength enhances technical stability in later stages. While the trunk does not directly generate propulsion, it plays a vital stabilizing role. The trunk enables limbs to exert greater force, even when their strength has limits. Given identical conditions such as height, weight, strength, aquatic sense, and technical framework, athletes with superior trunk control will invariably swim faster. This advantage becomes more pronounced as race distance increases. (C1)

C1's statement is particularly significant because it explicitly acknowledges that the trunk “does not directly generate propulsion” while still asserting its critical importance-a nuanced position that reconciles the direct and indirect accounts.

C5 warned that inadequate core strength can disrupt leg and stroke rhythm, particularly affecting sprint techniques. Here, the mechanism is not simply postural but rhythmic: core insufficiency disrupts the timing and coordination of limb movements.

In a 200 m front crawl event, technical stability often declines after the third 50 m, with increased lateral body swing during the final sprint. This is especially evident when athletes breathe unilaterally to the right, causing a noticeable rightward trunk bias as the left hand enters the water and catches up with the right hand’s power. This phenomenon, known as ’salute,’ can be technically detrimental even in athletes with strong core abilities during high-intensity competitions. With exceptional trunk strength, an athlete maintains body alignment, keeping the hips at the water’s surface throughout the swim, resulting in smooth movement. As the adage goes, ‘the trunk floats well. (C5)

The identification of the “salute” phenomenon-a specific technical fault linked to core insufficiency under fatigue-demonstrates the granularity of coaches’ observational knowledge.

C8 noted that under high lactate conditions, degradation of core control often manifests as breakdown in stroke rhythm and body position. Collectively, coaches viewed the core as an essential stabilizer that enables effective force application by the limbs, even if it does not directly generate propulsion. The distinction between direct and indirect mechanisms, along with representative coach quotations and links to the conceptual model, is summarized in Table 2.

**Table 2:** Direct and indirect mechanisms of core strength in front crawl performance: summary of expert coaches’ perspectives.

Mechanism	Definition	Performance pathway	Coach quotation	Observable outcome
Direct	Core actively generates or transmits propulsive force	Rotational power generation → Force transfer from trunk to limbs → Increased stroke propulsion	“Only the shoulders and hips engage in horizontal axis rotation, effectively transmitting power to the extremities”(C6)	Increased swimming speed; improved force output
Indirect	Core maintains alignment to reduce drag and enable limb efficiency	Trunk stabilization → Hydrodynamic alignment → Reduced resistive drag → Limb force preserved for propulsion	“Waist collapse..creates an ‘uphill swimming’ sensation.. increased resistance”(C5)	Maintained hip position; reduced lateral sway; delayed fatigue-induced breakdown

Direct mechanisms contribute actively to propulsion; indirect mechanisms preserve propulsive efficiency by minimizing energy loss through drag and postural compromise. Both pathways operate simultaneously, with relative importance varying by event distance, fatigue state, and individual athlete characteristics (see Theme 1.3, Threshold Effect).

#### 3.3.1 Threshold effect and individual variation

A minority of coaches cautioned against over-generalizing the importance of core strength, introducing an important qualification to the dominant narrative. C4 regarded it as one component within a broader performance equation, while C2 suggested that its significance is most apparent when addressing specific deficits in individual swimmers. Several coaches indicated a potential threshold effect, wherein improvements in core capacity may yield discernible performance gains mainly when baseline strength is suboptimal, as perceived by coaches.

Core stability and trunk strength training as integral to the overall training regimen, serving as a medium for showcasing athletic performance rather than a primary factor in its enhancement. Sports performance is multifaceted, and isolating the impact of a single factor requires extensive data. (C4)

C4’s cautionary note reflects a sophisticated understanding of performance determination-an awareness that multifactorial outcomes cannot be reduced to any single variable.

If an athlete’s weak core strength limits competitive ability, targeted training can significantly enhance body control, streamline posture to reduce resistance, and markedly improve performance. (C2)

C2's conditional statement (“if. limits”) explicitly frames core training as remedial rather than universally beneficial-a perspective with clear implications for assessment and prescription.

### 3.4 Theme 2: assessment of core strength

Coaches reported using a combination of land-based tests and water-based observations to evaluate core function. However, the relationship between these assessment modalities and their perceived validity reveals important insights into coaches’ epistemological orientations.

#### 3.4.1 Static and functional land tests

The static support test is widely regarded as effective, but coaches’ rationales for its use warrant examination. C3 emphasizes its importance in maintaining body posture during front crawl swimming, highlighting the necessity of “dynamic static support”-a term that captures the paradox of maintaining stability during movement. C4 views the plank support test as beneficial and low-risk, suggesting athletes should sustain support beyond competition durations. This recommendation implies a specific theory of transfer: that endurance in static holds predicts the ability to maintain posture under the fatigue conditions of racing.

However, side support is deemed risky due to potential spinal pressure, exceeding typical swimming loads. This concern reveals that coaches evaluate assessment tools not only for their diagnostic value but also for their risk profile-a consideration often absent from scientific discussions of testing.

C5 notes a scarcity of assessment methods, primarily utilizing static supports in various directions to gauge core endurance, considered fundamental. Notably, rotational ability is developed through upper-fixed weighted exercises yet remains untested in practice. This gap between training and assessment is significant: coaches are developing a capacity (rotational power) that they do not systematically evaluate.

#### 3.4.2 Video-based technical analysis

In-water video analysis was considered the most ecologically valid assessment tool. The reasoning underlying this preference is instructive: because swimming is unsupported, in-water analysis more accurately reflects the actual demands of the sport than land-based tests. C1 and C2 routinely used lateral and overhead recordings to identify technical flaws such as lumbar hyperextension (“sagging”) or lateral displacement of the hips. C6 used shoulder-hip alignment as a proxy for rotational efficiency, though C4 acknowledged the subjective nature of visual assessment.

Because swimming is unsupported, in-water analysis more accurately identifies weak trunk strength. Weak trunk strength is generally categorized into two types. The first is longitudinal weakness, often due to insufficient lumbar strength, causing the swimmer to arch their back. This can be assessed by observing the distance between the lumbar depression and the horizontal plane via side-view filming. The second type is lateral weakness, leading to side-to-side swaying. This is detectable by measuring the amplitude of bilateral swaying through top-view filming to identify the weaker side. (C1)

C1's description is remarkable for its specificity: he has developed an implicit coding scheme that categorizes core insufficiency into two types (longitudinal/lateral) and links each to specific observable indicators. This represents a form of tacit knowledge that has been refined through years of observation.

An athlete’s core strength is best evaluated by their in-water performance. While physical fitness tests have some utility, they do not directly correlate with swimming performance improvement and are not decisive.In-water technical analysis is crucial and often relies on a coach’s intuition. (C2)

C2's reference to “intuition” is significant-it acknowledges the role of tacit, pattern-recognition knowledge that cannot be reduced to formal metrics. This does not represent a weakness in coaching practice but rather the exercise of expertise developed through extensive experience.

#### 3.4.3 Integrated and sport-specific evaluation

Some coaches noted that alternative testing methods can enhance the assessment of athletes’ core strength. C6 asserted that the erector spinae muscles are crucial for maintaining balance in water, linking this to the Functional Movement Screen (FMS).

The Functional Movement Screen (FMS) test, which includes trunk stability and rotational stability assessments, effectively identifies athletes’ imbalance issues. Many injuries stem from muscle strength imbalances or weaknesses on one side. Addressing these imbalances by strengthening relevant muscles is seen as a solution. The trunk rotation skills in the front crawl swimming event is very important particularly in exercises like trunk rotation ball-hitting and prone cable row. (C6)

C7 also supported the FMS for its diagnostic value in evaluating athletes. C1 advocated for a holistic approach that combines trunk stability, power, and inter-segmental coordination. He noted that land-based strength does not always transfer directly to water and stressed the need for task-specific evaluation.

Athletes’ abilities should be evaluated holistically, encompassing trunk stability, explosive power, and coordination between the trunk and upper limbs, exemplified by the rotational pulling force in front crawl swimming. The difficulty in effectively integrating these abilities with the force generated by the lower limbs. (C1)

C1's emphasis on integration and coordination-rather than isolated capacities-reflects an understanding of the core as part of a dynamic system rather than a collection of independent abilities.

### 3.5 Theme 3: core training practices

Coaches described a spectrum of training strategies aimed at improving core capacity, with an emphasis on specificity and transferability. Based on coach recommendations, core strength training for front crawl swimmers was categorized into four components (Table 3): increasing land-based core strength reserves is the prerequisite, improving in-water core stability is the foundation, strengthening land-to-water transfer is the key, and refining technical movements is the core.

**Table 3:** Key components of core training for front crawl swimmers.

Training component	Description	Coach examples
Land-based strength building	Developing core strength reserves through various exercises	Planks, compound lifts, medicine ball throws
In-water stability training	Enhancing sport-specific core stability in aquatic environment	Resisted pulling, weighted kicking drills
Land-to-water transfer	Bridging the gap between dry-land and aquatic performance	Integrated drills, technical progression
Technical refinement	Fine-tuning movement patterns and force application	Single-arm drills, proprioceptive training

Examples are drawn from multiple coaches’ accounts; see text for specific attributions and contextual details.

#### 3.5.1 Increasing land-based core strength reserves

Developing athletes’ core strength first requires strengthening land-based training to build core strength reserves. The importance of land training is widely recognized, but the reasoning behind this priority is instructive: land training provides a foundation of capacity that can then be expressed in the water.

As C1 stated, “*The primary goal is to establish a solid core strength platform through land training, improving peak maximum strength or relative strength endurance, which can then be better expressed through specialized training.*” He summarized this as “*land training builds the platform and height, while specialized training manifests the breadth.*”

This platform metaphor is analytically significant: it frames land training as building generic capacity, while water training develops the specific coordination patterns that enable that capacity to be expressed in performance.

Regarding the developmental sequence, C5 proposed first establishing a land-based foundation with an emphasis on the muscular endurance of the abdominal and back stabilizers through multi-directional static holds. This initial phase is followed by integrated whole-body power development. Training-including dynamic/static holds, medicine ball work, and KEISER exercises-is periodized and typically scheduled after water sessions to avoid interference with high-intensity swimming.

While static holds are a common training method, coaches emphasize different aspects. C5 valued them as an indicator of core maturity and technical stability, noting the disparity in endurance between mature and young athletes. C3, however, prioritized resisted static holds or dynamic, anti-interference stability, and observes that dynamic training integrated with limb movement is more prevalent in practice.

For building maximum strength, coaches highlighted compound lifts; C3 and C4 specified that exercises like squats, deadlifts, and the hip thrust 1RM not only build strength but also enhance spinal stability via intra-abdominal pressure. This understanding-that compound lifts contribute to core function through mechanisms beyond direct muscle activation-reflects sophisticated biomechanical knowledge.

Conversely, C6 deemed rotational power-trained with high-intensity medicine ball throws-as most critical, followed by torso strength endurance developed on unstable surfaces.

For example, kicking while supported on a Swiss ball requires core stability; performing the kick under unstable support demands controlled, whip-like stabilization. Holding onto a bar and performing butterfly kick towards a ball, but controlling lumbar engagement to manage amplitude and gradually improve rhythm. (C6)

This example illustrates the principle of progressive overload through instability-a concept that bridges land and water training by introducing an unstable base that simulates the aquatic environment.

#### 3.5.2 Enhancing in-water core stability training

C1 considered resisted water pulling exercises the primary method for enhancing sport-specific core stability. This includes using equipment like metal plates, buckets, or resistance bands tied to the waist to provide load and challenge core stability. Secondly, in-water weighted training, such as using a weighted belt for arm pulls with legs immobilized, is also common.

As C1 explained, “*Including pulling drills with legs bound (no kickboard) controls the lower body. Using a kickboard makes the lower body float up, so this method improves control over the lower half. Preventing kicking is itself a form of core control training.*”

This insight is significant: C1 explicitly identifies the removal of a supportive device (kickboard) as a core challenge, revealing an understanding that core control is often masked by compensatory strategies.

He also detailed methods for in-water core strength training, such as progressively adding weight to the waist while performing kicks in four directions (prone, supine, left, right) and lateral swimming. Progression moves from front crawl to backstroke, sidestroke, and lateral swimming, “*because sidestroke involves kicking without arm movement, making it harder to control the front, thereby increasing demands on lumbar stability*.”

Based on stroke, in-water core training is divided into two types: stability training for front crawl and backstroke, and undulation training for butterfly, breaststroke, and underwater dolphin kick. In practice, combining weighted waist training with various kicking forms enhances overall athlete capacity.

“For instance, alternating front crawl and dolphin kick improves dynamic, static, rotational, and contractile qualities. Training should be hip-driven, involving rotation or undulation. This includes front crawl kick with waist weight, or adding ankle resistance like drag socks, performing kicks in four positions (prone, left side, right side, supine). Alternating dolphin kick and front crawl kick is also used. Furthermore, performing vertical kicking in the water with waist weight, and as ability improves, gradually raising the arms or increasing the overhead load. To break the water surface, the athlete must exert more force, requiring contribution from the waist/undulation or lumbar control—this is how improvement happens.” (C1)

This extended description reveals a sophisticated, multi-dimensional training system that systematically challenges core function across different postures, movement patterns, and resistance levels.

#### 3.5.3 Strengthening land-to-water transfer training

C3 pointed out that many athletes possess good physical fitness but struggle to effectively control body position or generate propulsion in the water, resulting in slower speeds—an issue present even among some elite performers, though less pronounced. This observation identifies the transfer problems a central challenge in core training.

Addressing this, C2 emphasized the need for extensive integrated land-water training:

“It must be practical; some things trained on land must be manifested in the water. Some individuals have excellent core strength on land but cannot translate it underwater. This requires substantial integrated land-water training, which also involves ‘feel for the water’. This integration is currently paramount. Many of our athletes show rapid strength gains on land, but their feel in the water lags significantly. Increased muscle mass can also impact their training and competitive performance.” (C2)

C2's reference to “feel for the water” introduces a perceptual dimension to transfer: the athlete must not only have the strength but also develop the sensory awareness to deploy it effectively in the aquatic environment. Coaches’ emphasis on ‘feel for the water’ aligns with ecological approaches to motor learning, where perception–action coupling is critical for transfer.

C7 believed that training in water is closer to competition reality; all land training should serve the water. Transferring land-based capacity into the water is a process:

“This is very important. Often, after performing certain movements on land, many athletes cannot replicate them in the water—they can't do it, they lack the feel. Therefore, we need to use in-water technical guidance and drills, allowing them to gradually discover that sensation before transferring it to full-stroke coordination.” (C7)

The emphasis on “discovering that sensation” highlights the role of proprioceptive learning in transfer-a dimension often overlooked in purely biomechanical accounts. The identified gap between land-based strength and water-based performance reflects the principle of specificity (27) and suggests that core training must account for task constraints, not just muscle activation.

#### 3.5.4 Refining technical movements

C5 indicated that in-water drill training is an excellent method for coordinating force application. Simplifying movements and reducing difficulty allows for better mastery of force production. The first step is breaking down the technique with the athlete, starting with basics like specific kick and pull drills, before combining them.

“It also requires extensive practice. After mastering the components, integrating them feels different. Start with simpler drills, including the many land-based training methods available now, which are quite integrated with water training patterns.” (C5)

This description reflects a classic part-whole learning approach, adapted to the specific demands of core-integrative swimming skills.

Coach C5 viewed the application of core strength in technique as a matter of practice making perfect, requiring frequent training, especially given the high demands of swimming in this regard. He also noted that athlete technique aims for faster swimming, where high-quality technique and stroke efficiency require stability. Only by linking limb strength with core strength can overall capacity improve and technical stability be enhanced. Otherwise, the body cannot execute the technique, even if learned.

C7 believed that after using land training to guide athletes in torso rotation (e.g., simulating the water state on a Swiss ball with instability), in-water training should employ more drills. These include single-arm drills (one arm forward, one back) and various torso rotation exercises helping athletes locate the ‘X’ crossover point of force application during swimming. Using a snorkel can help establish water feel before focusing on force at the crossover point.

“First, rotate the torso; a side-lying starting position is needed. Using a snorkel eliminates breathing concerns, allowing them to focus on feeling the force application point at the crossover. A stable head helps sense and locate this point. Using paddles and fins serves a similar purpose.” (C7)

This type of proprioceptive training for specific techniques stimulates the nervous system, muscle memory, and body awareness. Through continuous breakdown and gradual mastery, a complete, coordinated force application pattern develops.

“When this feeling is lost during full stroke, it’s essential to return to drills, starting from the beginning to rediscover the core force application point.. The core acts as a bridge for transmission. Previously, it might not have been considered very important, but subsequent research has shown its significant impact on sports performance. This is a crucial aspect. Even at high levels and older ages, finding new sensations through fine-tuning is necessary for performance breakthroughs.” (C7)

C7’s description of returning to drills when the feeling is lost reveals an understanding of core training as an ongoing process of refinement rather than a one-time capacity-building endeavor.

### 3.6 Synthesis: towards a conceptual model

Across the three themes presented above, a coherent picture emerges of how expert coaches conceptualize, assess, and train core strength for front crawl performance. Coaches perceive the core as serving dual functions-direct (force transmission, rotational power) and indirect (stabilization, drag reduction)-with the relative importance of each mechanism varying by athlete and context. Assessment practices reveal a tension between the perceived validity of land-based tests and the ecological validity of in-water observation, with coaches privileging the latter based on their experiential knowledge that transfer cannot be assumed. Training practices emphasize a sequential, integrated approach: building land-based strength reserves, enhancing in-water stability, facilitating land-to-water transfer, and refining technical execution.

However, the coaches’ accounts also reveal two critical moderating factors that shape the effectiveness of core training: individual athlete characteristics (baseline core capacity, technical proficiency) and contextual factors (event distance, fatigue state). The identification of a potential threshold effect-wherein core training yields discernible gains primarily when baseline strength is suboptimal-is particularly significant, as it offers a framework for reconciling conflicting findings in the scientific literature.

**Figure 2:**
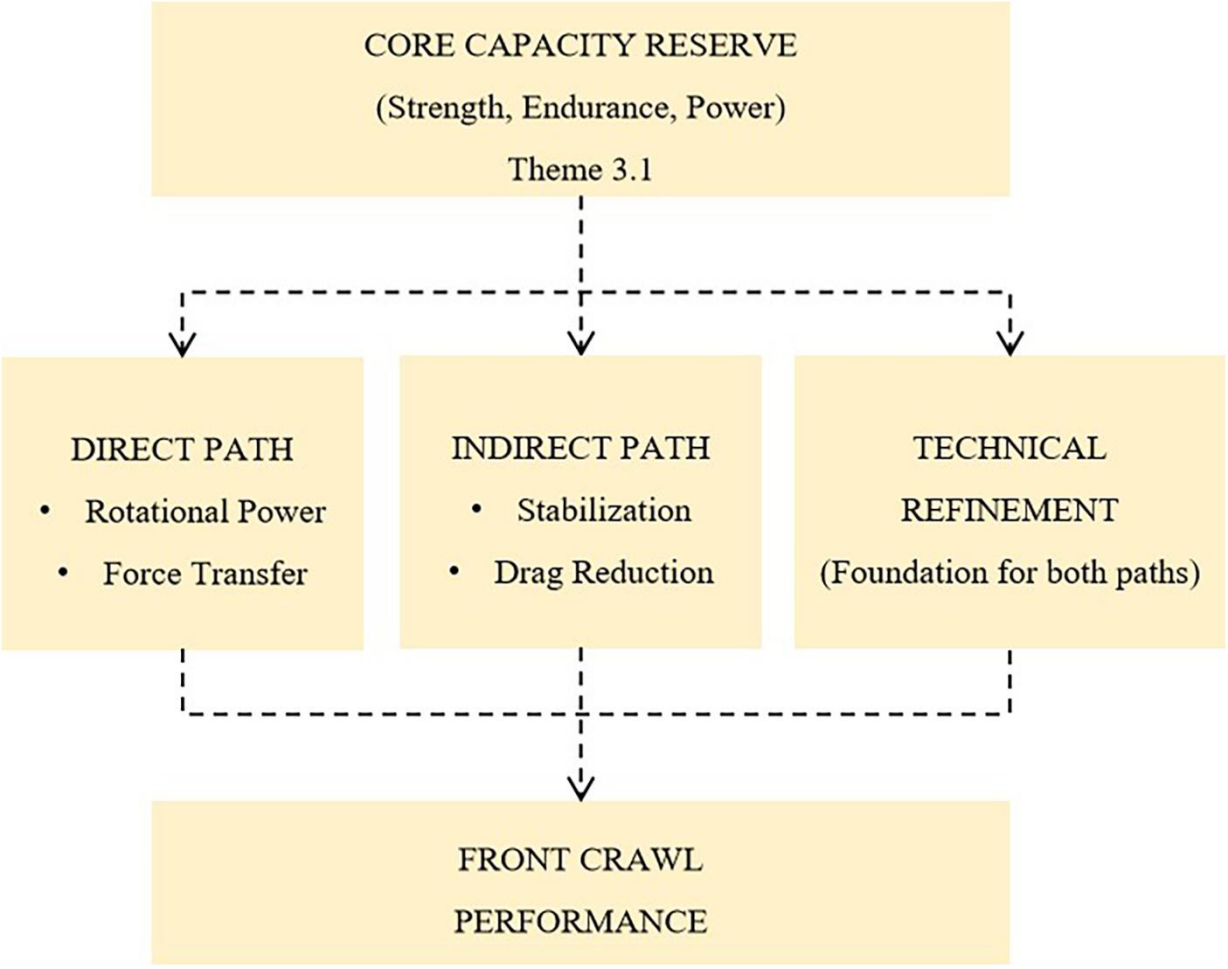
A conceptual model of core strength's role in front crawl performance: synthesis of expert coaches’ perspectives. This model synthesizes coaches’ experiential knowledge, proposing that land-based core capacity (Themes 3.1) influences front crawl performance through direct (force transmission, rotational power) and indirect (stabilization, drag reduction) pathways (Themes 1.2). Two factors moderate the translation of core capacity into performance. First, a ‘threshold effect’ (Theme 1.3): core training may yields discernible gains primarily when baseline capacity is below a functional threshold. Second, ‘transfer efficacy’ (Theme 3.3): land-based strength must be deliberately coupled with water-specific drills to be effective. Technical refinement (Theme 3.4) underpins both pathways. The model is a hypothesis-generating framework derived from coaches’ perceptions, not an empirically validated causal pathway. Elements are traceable to thematic findings (Themes 1.2, 1.3, 3.1, 3.3, 3.4).

The synthesis of these findings-the mechanisms, the practices, and the moderators-points toward an integrative framework that can guide both future research and coaching practice. This framework is offered as a hypothesis-generating tool rather than a validated causal model. It is presented as a conceptual model in the Discussion (Figure 2), with each element explicitly linked to the thematic findings that informed it. Table 4 summarizes the key thematic findings and their contributions to the conceptual model.

**Table 4:** Summary of thematic findings and their contribution to the conceptual model.

Theme	Key findings	Contribution to model
Theme 1: Functional Role	Dual mechanisms (direct/indirect); threshold effect; individual variation	Identifies pathways linking core to performance; introduces moderating variables
Theme 2: Assessment	Preference for ecological validity; gap between trained and assessed capacities	Highlights need for sport-specific assessment; reveals reliance on experiential knowledge
Theme 3: Training Practices	Sequential approach; emphasis on transfer; progressive overload through instability	Informs intervention design; identifies key training principles

## 4 Discussion

This qualitative study aimed to examine how expert high-performance swimming coaches perceive the role of core strength in front crawl performance, specifically exploring their conceptualizations, assessment practices, and training methodologies. The analysis yielded three principal findings. First, despite variations in how they anatomically defined the core, all coaches unanimously affirmed its functional importance, describing both direct (rotational power, force transmission) and indirect (stabilization, drag reduction) mechanisms through which core strength influences performance. Second, a significant theory-practice gap emerged: while coaches held strong beliefs about core function, their assessment and training practices relied heavily on subjective observation and anecdotal experience, reflecting the absence of validated, swimming-specific diagnostic tools. Third, coaches’ experiential knowledge pointed toward a threshold effect-whereby core training may yield more noticeable gains in athletes with initial deficiencies-and emphasized the critical importance of land-to-water transfer. In the sections that follow, we discuss each finding in relation to existing evidence, interpret key findings through theoretical lenses (motor learning, specificity, kinetic chain), and propose a hypothesis-generating conceptual model.

The coaches’ identification of a dual-mechanism model offers a pragmatic framework for reconciling inconsistent findings in the literature. The indirect mechanism aligns with biomechanical principles of drag reduction (7), as coaches vividly illustrated through descriptions of core insufficiency leading to observable technical faults—such as “waist collapse,” lateral sway, and the “salute” phenomenon—that increase drag and compromise efficiency. The proposition of a direct propulsive contribution challenges a purely stabilizing paradigm (15) and directs attention to the trunk as a kinetic link. This aligns with emerging evidence on the role of trunk rotation in generating propulsive forces during front crawl (10, 15), suggesting that the core may contribute to propulsion not only through stabilization but also through active force generation during rotational movements. This perspective necessitates a re-evaluation of training modalities, moving beyond stability-endurance drills to include dynamic, power-oriented exercises that mirror the coaches’ emphasis on rotational strength (11, 12, 28). The coexistence of these two mechanisms in coaches’ accounts suggests that core function in front crawl is not an either/or proposition but rather a context-dependent integration of both pathways, with relative importance varying by athlete characteristics and situational factors. While coaches’ perceptions provide ecologically valid insights, their mechanism claims should be treated as hypotheses for future studies combining kinematic analysis, electromyography, and performance testing. Existing biomechanical studies (10, 15) provide partial support, but systematic integration of coach perceptions with laboratory-based measures remains lacking.

A central, and perhaps the most significant, finding of this study is the stark contrast between the coaches’ unanimous belief in core strength and the absence of systematic, evidence-based methods for its assessment and development. This practice-theory paradox manifests in a heavy reliance on subjective video analysis and anecdotal experience. The root of this paradox appears to lie in a critical methodological void: the lack of standardized, ecologically valid assessment tools that can translate land-based strength metrics into swimming-specific performance (29, 30). Coaches intuitively bypassed tests with poor face validity (e.g., prolonged static holds), instead favoring in-water technical observation to evaluate core function in context. This preference should not be dismissed as mere resistance to scientific methods; rather, it reflects a sophisticated appreciation for ecological validity. As C2 noted, “physical fitness tests.. do not directly correlate with swimming performance improvement and are not decisive.” Coaches have developed implicit coding schemes (e.g., C1's distinction between longitudinal and lateral weakness) that enable them to diagnose core insufficiency through observable technical flaws. This practitioner wisdom highlights a fundamental limitation in the current sport science literature and calls for the co-creation, with coaches, of new diagnostic protocols that are both scientifically rigorous and practically applicable.

Three theoretical lenses help interpret key findings beyond description. First, ecological motor learning. Coaches’ emphasis on “feel for the water” aligns with ecological approaches, where perception–action coupling is critical for transfer. The inability to replicate land-based strength in water-described by C7 as “they can't do it, they lack the feel”-reflects a breakdown in the perception–action cycle that sport-specific drills aim to repair. Second, specificity of training. The gap between land-based strength and water-based performance reflects the specificity principle (27). Core training must account for task constraints-the unstable, rotational, fatigue-inducing demands of swimming-not just muscle activation patterns. Generic core endurance tests (e.g., static planks) have limited predictive validity precisely because they violate specificity. Third, kinetic chain principles. Coaches’ descriptions of “waist collapse” and “salute phenomenon” illustrate how proximal instability disrupts distal force application. From this perspective, the core is not an independent contributor but a link whose dysfunction cascades through the system, forcing compensatory movements that degrade efficiency.

The threshold effect perceived by coaches provides a plausible explanation for the muted effects often seen in intervention studies on elite swimmers (14, 15). If confirmed empirically, this would suggest a functional threshold beyond which generic core training yields diminishing returns, necessitating more specialized stimuli. This has significant implications for study design: interventions failing to account for baseline core function may obscure true effects by averaging across athletes above and below the threshold. Furthermore, the overarching principle of integrated land-water training emerged as a cornerstone of effective prescription. Coaches consistently viewed land training as a means to build a “strength reservoir,” the benefits of which are only realized through targeted water-based drills that facilitate transfer. This practice is firmly grounded in the principle of training specificity (27, 31), yet its application in core training has been limited in the scientific literature. Coaches’ emphasis on transfer reflects an implicit understanding that core capacity developed in stable land environments does not automatically translate to the unstable, rotational demands of front crawl. The success of combined training regimens, as an example, provides preliminary evidence for this approach (11, 28, 32), underscoring the need for future research to not just isolate variables but to optimize the complex interplay between different training modalities.

Synthesizing the thematic findings, we propose a conceptual model (Figure 2) integrating coaches’ perspectives on how, when, and for whom core strength influences front crawl performance. The model posits that land-based core capacity (Theme 3.1) enhances performance through direct and indirect mechanisms (Theme 1.2), moderated by a threshold effect (Theme 1.3) and land-to-water transfer efficacy (Theme 3.3), with technical refinement (Theme 3.4) underpinning both pathways. The model is offered as a hypothesis-generating tool, not a validated causal framework. Each element is traceable to thematic findings from the Results section. The following testable hypotheses emerge from the model: (1) core training interventions will show larger effects in athletes with core deficiencies (below-threshold) compared to those with adequate baseline function; (2) integrated land-water training protocols will produce superior transfer compared to isolated approaches; and (3) the relative contribution of direct vs. indirect mechanisms will vary by event distance—sprint performance relying more on rotational power, distance performance on fatigue resistance and postural maintenance.

The identified assessment–training gap has three concrete implications. First, coach education. Programs should explicitly address how to systematically observe and classify core-related technical faults (e.g., longitudinal vs. lateral weakness) using video analysis frameworks co-developed with practitioners. Coaches like C1 have already developed implicit coding schemes; formalizing these could enhance diagnostic consistency. Second, assessment tool development. Sport scientists should prioritize developing swimming-specific core tests that are both reliable and ecologically valid—such as in-water rotational power tests, fatigue-induced postural change metrics (e.g., hip drop during repeated sprints), and instrumented kickboard protocols. Third, targeted communication of research findings. Research findings should be communicated with explicit guidance on “for whom and under what conditions.” The threshold effect suggests generic core training may be unnecessary for athletes already above the functional threshold, whereas it may be transformative for those below it. For practitioners, the model offers a framework for individualizing prescription: assess baseline function relative to threshold, target athletes with deficiencies, design for explicit land-water transfer, and recognize technical refinement as integral to accessing core capacity.

This study has several limitations that warrant consideration. First, generalizability. The sample of eight expert Chinese coaches reflects a specific cultural and educational context (33). Transferability to other coaching cultures requires empirical investigation. The underrepresentation of female coaches (*n* = 1) reflects the gender composition of high-performance swimming coaching in the Chinese context. Second, confirmation bias. The unanimity of responses may partly reflect a common coaching discourse rather than fully independent perspectives. The interviewer's background as an elite conditioning coach—while facilitating rapport—may have inadvertently reinforced certain assumptions about core strength. Although safeguards were implemented (independent initial coding, team reflexivity), readers should interpret consensus findings with this caveat. Third, self-report data. Reliance on self-report interviews does not capture divergences between reported and actual practice-a gap observational studies could address. Fourth, interview duration. Interview durations (22–35 min) were constrained by coaches’ schedules. While data saturation was achieved, longer or multiple interviews might have yielded deeper exploration. Fifth, member checking was not conducted due to coaches’ availability constraints, limiting participant verification of interpretations. Sixth, the conceptual model remains hypothetical and requires testing through intervention studies incorporating athlete-level moderators. The absence of objective performance data means we cannot determine whether coaches’ beliefs align with measurable outcomes—a priority for future mixed-methods research. Seventh, the focus on front crawl limits applicability to other strokes, which may impose different core demands (e.g., butterfly's trunk flexion-extension vs. front crawl's rotational stability).

## 5 Conclusion

This study transcends merely confirming the importance coaches place on core strength. It delineates the specific mechanisms they value, exposes the methodological challenges that perpetuate a reliance on experience, and puts forward testable concepts derived from practice. The journey toward evidence-based core conditioning in swimming requires a collaborative bridge between the locker room and the laboratory. By taking the experiential knowledge of expert coaches as a starting point, sport scientists can design more relevant and impactful research, ultimately working toward a more nuanced and testable framework for performance enhancement.

## Data Availability

The original contributions presented in the study are included in the article/Supplementary Material, further inquiries can be directed to the corresponding author.
